# A new predator connecting the abyssal with the hadal in the Kuril-Kamchatka Trench, NW Pacific

**DOI:** 10.7717/peerj.4887

**Published:** 2018-06-07

**Authors:** Anne-Nina Lörz, Anna Maria Jażdżewska, Angelika Brandt

**Affiliations:** 1 Centre of Natural History, Zoological Museum, University of Hamburg CeNak, Hamburg, Germany; 2 Laboratory of Polar Biology and Oceanobiology, Department of Invertebrate Zoology and Hydrobiology, Faculty of Biology and Environmental Protection, University of Lodz, Lodz, Poland; 3 Department of Marine Zoology, Section Crustacea, Senckenberg Research Institute and Natural History Museum, Frankfurt am Main, Germany; 4 Institute for Ecology, Evolution and Diversity, Goethe-University of Frankfurt, Frankfurt am Main, Germany

**Keywords:** Rhachotropis, Amphipoda, Hadal, Kuril-Kamchatka trench, Abyssal, Integrative taxonomy

## Abstract

The bathyal to hadal deep sea of north-west Pacific Ocean was recently intensively sampled during four international expeditions (KuramBio I and II, SoJaBio and SokhoBio). A large amphipod, *Rhachotropis saskia* n. sp., was sampled in the Kuril-Kamchatka Trench and increases the number of described hadal species of that area to eight. A detailed description of the new species is provided, including illustrations, scanning-microscope images and molecular analysis. This predatory species was sampled at both continental and ocean abyssal margins of the Kuril-Kamchatka Trench as well as at hadal depths of the trench. The wide bathymetric distribution of the new species over more than 3,000 m is confirmed by molecular analysis, indicating that the Kuril Kamchatka Trench is not a distribution barrier for this species. However, the molecular analysis indicated the presence of isolation by distance of the populations of the studied taxon.

## Introduction

Exploring hadal depths has been vitalised in the last decade (Jamieson, 2015). The focus of biological research at these extreme depths has been the deployment of baited cameras and traps, resulting in an increased knowledge of snailfish and scavenging amphipods (Jamieson et al., 2011; Lacey et al., 2017). The recent increases in sampling effort at hadal depths over extensive bathymetric ranges and across several trenches and the fixation of material suitable for molecular analyses now allow studies at both intra- and inter-trench levels (Fujii et al., 2013; Lacey et al., 2016; Ritchie, Jamieson & Piertney, 2016; Devey & Shipboard scientific party, 2015; Brandt et al., 2015a; Brandt et al., 2015b; Brandt & Shipboard scientific party, 2016). Hadal amphipods have been the subject of numerous and diverse studies in recent years (Blankenship et al., 2006; Blankenship & Levin, 2007; Jamieson et al., 2011; Kobayashi et al., 2012; Eustace et al., 2013; Eustace et al., 2016; Ritchie, Jamieson & Piertney, 2015; Ritchie, Jamieson & Piertney, 2016; Lacey et al., 2016; Lacey et al., 2017). However, all these amphipod studies focus on scavenging amphipods, as these are the animals easily collected in large numbers via baited traps.

Since the 1950-ties, the Kuril-Kamchatka Trench (KKT) area was intensively sampled during several oceanographic cruises on the RV *Vitjaz.* These studies resulted in the identification of 48 deep-sea amphipod species from that area; of these 16 benthic species were sampled below 3,000 m (Birstein & Vinogradov, 1955; Birstein & Vinogradov, 1958; Birstein & Vinogradova, 1960; Kamenskaya, 1981; Kamenskaya, 1997; Shimomura & Tomikawa, 2016). Only seven of them were found in the hadal zone of Kuril-Kamchatka Trench. However, it was suggested that the actual diversity of these waters is largely underestimated, as the gear used during *Vitjaz* cruises were suitable to collect larger macrofauna and megafauna, while smaller macrofaunal invertebrates were not caught at all (Birstein, 1963). Additionally the recent molecular studies revealed that in several cases widely distributed nominal species are in fact species complexes of much narrower geographic or bathymetric ranges (e.g., Havermans et al., 2013; Brix, Svavarsson & Leese, 2014). Unfortunately, the extensive material collected during *Vitjaz* expedition is not suitable for genetic studies.

In recent years, two German-Russian expeditions to the Kuril-Kamchatka Trench and adjacent abyssal plain with RV *Sonne* were conducted (Kurile-Kamchatka Biodiversity Studies, KuramBio I and II, 2012 and 2016). These expeditions together with another two Russian-German cruises: SoJaBio (Sea of Japan Biodiversity Studies) in 2010 and SokhoBio (Sea of Okhotsk Biodiversity Studies) in 2015, aimed to study the biodiversity and biogeography as well as trophic characteristics of the benthic organisms in these different northwest Pacific deep-sea environments (Malyutina & Brandt, 2013; Brandt & Malyutina, 2015; Brandt & Shipboard scientific party, 2016; Malyutina & Brandt, 2018). The samples were collected using a variety of equipment according to standardised protocol allowing extensive comparative collections of invertebrates from all deep-sea habitats, suitable also for molecular studies.

Since the proposal of the DNA barcoding concept by Hebert, Ratnasingham & DeWaard (2003) the use of molecular methods in species identification has become very popular (e.g., Wang et al., 2018 and references therein) and a genetic approach is often used to supplement morphological taxonomy (e.g., Hubert & Hanner, 2015; Seefeldt et al., 2017). The use of more than one type of character in species recognition becomes the basis of the increasingly more common integrative taxonomy. However, Pante, Schoelinck & Puillandre (2015) underlined that only a small number of papers that have used molecular methods in species delineation revealing the existence of new species were accompanied with their formal descriptions. The use of molecular markers has highlighted the existence of many overlooked species within the Order Amphipoda both in freshwater as well as marine environments (e.g., Lörz et al., 2009; Havermans et al., 2013; Mamos et al., 2016; Verheye, Backeljau & d’Udekem d’Acoz, 2016). Therefore, the studies of the deep-sea invertebrate fauna employing genetic methods have become popular in the recent years (Taylor & Roterman, 2017) including publication of the mitogenome of the amphipod *Hirondellea gigas* collected from the Challenger Deep of Mariana Trench (Lan et al., 2016). But apart from the contributions presenting different “World Records” (like the paper cited above) there are still not many studies using genetics for deep-sea invertebrate biogeographic analyses, especially if concerning abyssal and hadal depths (Taylor & Roterman, 2017). In comparison to 400 known deep-sea amphipod species only ten of them have wide geographic distribution (Brandt et al., 2012; Jażdżewska, 2015). However, the isolation-by-distance (IBD) analysis on the available data suggest that the deep-sea invertebrates have larger species ranges than their shallow water counterparts (Baco et al., 2016) and that diversity decreases between bathyal and abyssal depths (source–sink hypothesis) (Rex et al., 2005; Taylor & Roterman, 2017). The population connectivity between abyssal and hadal zones at the molecular level was not yet studied.

Both KuramBio expeditions sampled 39 stations in the depth range 4,900–9,500 m. Crustaceans, including amphipods, constituted an important component of collected invertebrates in terms of abundance (Brandt et al., 2015a; Brandt et al., 2015b; Brandt & Shipboard scientific party, 2016). Among amphipod crustaceans frequently encountered were the individuals of the family Eusiridae (represented mainly by the genus *Rhachotropis*) (Jażdżewska, 2015; Golovan et al., 2018).

The genus *Rhachotropis* S.I. Smith, 1883 (Eusiridae) contains 62 species (Horton et al., 2018). *Rhachotropis* is found in all oceans, and has the greatest bathymetric distribution (0–7,160 m) among all amphipod genera (Lörz et al., 2012). Some *Rhachotropis* species are known to be abundant locally at bathyal depths (Cartes & Sorbe, 1999) but increased deep-sea sampling shows *Rhachotropis* also to be amongst the most dominant amphipods in abyssal depth.

The identification of an amphipod collection from the Kuril-Kamchatka area obtained at several stations from different depths revealed one species new to science. We herein describe this species in detail and attempt testing if horizontal and/or vertical distances influence it’s genetic diversity.

## Material and Methods

### Study area

**Figure 1:**
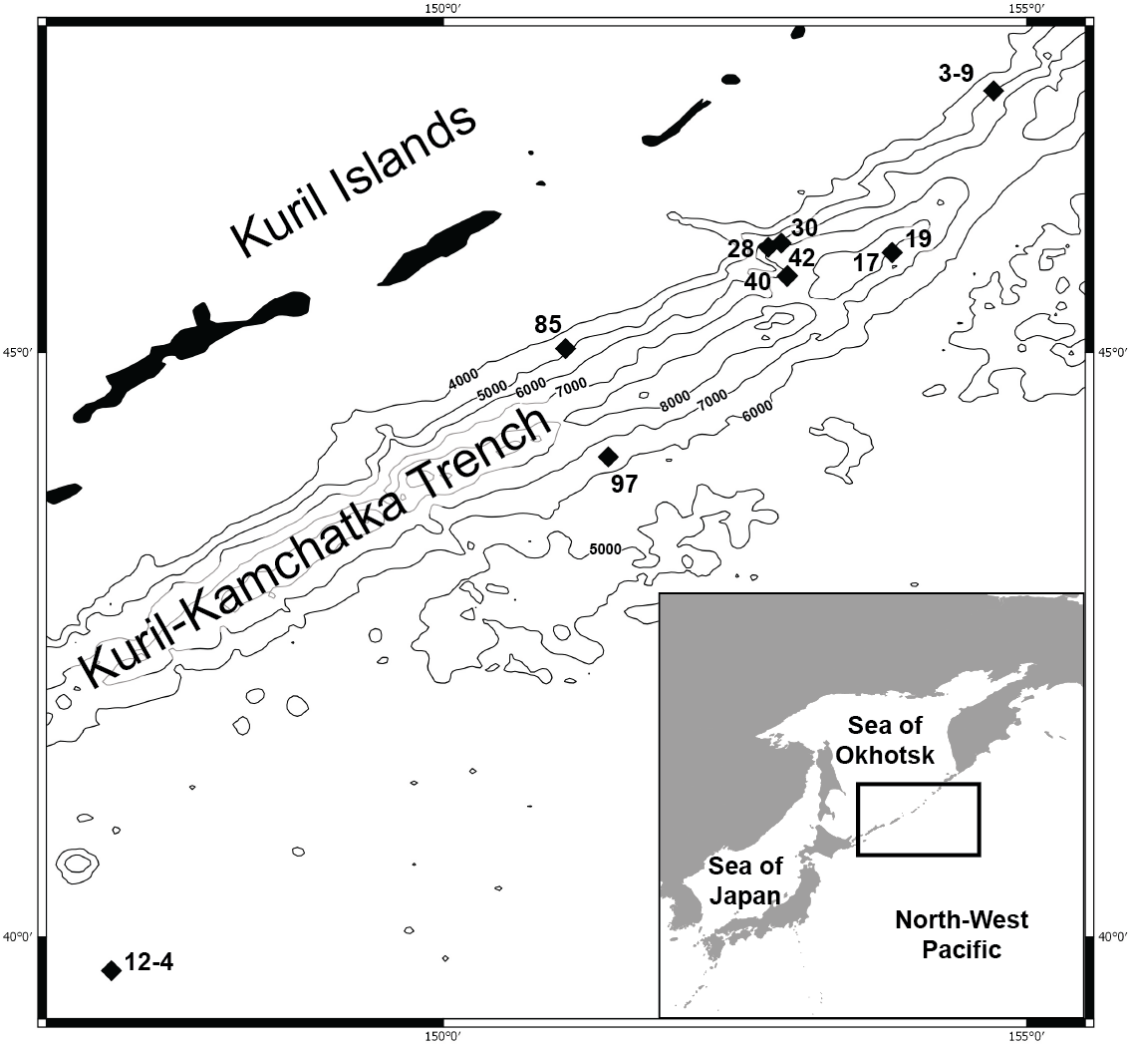
Map of the Kuril Kamchatka Trench sampling area. Sampling stations are indicated with diamonds.

The Kuril–Kamchatka Trench (KKT) (Fig. 1) is an oceanic trench in the northwest Pacific Ocean and extends from the southeast coast of Kamchatka in parallel to the Kuril Island chain to meet the Japan Trench east of Hokkaido. The trench formed as a result of the subduction zone, which formed in the late Cretaceous and has created the Kuril island arc as well as the Kamchatka volcanic arc. The Pacific Plate is being subducted beneath the Okhotsk Plate along the trench, resulting in intense volcanism (Malyutina & Brandt, 2018; Belousov et al., 2015).

The hydrography of the KKT area is complex. The main hydrological properties are different in its western part compared to the eastern side of the KKT. The thermohaline characteristics of the water of the Kuril Current show that surface water temperature ranges from 4–8 °C at the north-western side to the direction of the Sea of Okhotsk, whereas in the counter-current the temperature ranges from 5–13 °C (Arseniev & Leontieva, 1970). Deep flows on the slope inshore of the KKT southeast off Cape Erimo, Hokkaido, show that the lower flows (>3,000 m) are controlled by the local bottom topography and form in part a southward deep western boundary current (Uehara & Miyake, 1999). The bottom currents in this area are derived from the northward flowing Lower Circumpolar Deep Water (Kawabe & Fujio, 2010; Takeuchi, Tomikawa & Lindsay, 2016). Drifter Observations of Anticyclonic Eddies near Bussol’ Strait allowed to recognize spatial variations in the presence of a warm-core which originated as a Kuroshio ring and propagated to the vicinity of the Bussol’ Strait from the south, but also refer to a cold-core eddy which was apparently generated locally near Bussol’ Strait by an intensive supply of low potential vorticity water outflowing through the strait from the Okhotsk Sea (Rabinovich, Thomson & Bograd, 2002).

### Sampling

The samples were taken by a camera-epibenthic sledge (C-EBS) at abyssal depths (Brandt et al., 2013; Brandt et al., 2015a; Brandt et al., 2015b) and an epibenthic-slege (EBS) (Brenke, 2005) at hadal depths. Both gears are equipped with supra- and epibenthic samplers possessing two plankton nets (500 µm) on top of each other leading to two cod ends (300 µm). All samples were fixed in precooled (−20 °C) undenatured 96% ethanol and treated as described in Riehl et al. (2014). Large amphipod specimens were immediately sorted on deck, fixed in −20° precooled 98% ethanol and later transferred to 96% ethanol. Amphipod samples collected between 3,000 m and 6,000 m are regarded as “abyssal”, deeper than 6,000 m as “hadal” following the classification of abyssal amphipods by Barnard (1962).

Sampling permission was provided via Russia, port authority, permission 49 from 5.4.2016.

### Morphological description

Specimens were examined and dissected under a Leica MZ12.5 stereomicroscope and drawn using a camera lucida. Small appendages (mouthparts) were temporarily mounted in glycerin and examined and drawn using a LeicaDM2500 compound microscope fitted with a camera lucida. The body lengths of specimens examined were measured by tracing individual’s mid-trunk lengths (tip of the rostrum to end of telson) using a camera lucida. All illustrations were digitally ‘inked’ following Coleman (2003), Coleman (2009).

One adult paratype was dried, coated with gold-paladium and investigated using a scanning electron microscope LEO1525.

Photographs were taken with a Canon EOS 5 Mark III with lens Canon MP-E65 macro mounted for stacking; the stacking programme software is Zerene Stacker 1.04 (setting P-max) at the Centre of Natural History (CeNak).

The type material is held at the Senckenberg Museum Frankfurt, Germany.

The electronic version of this article in Portable Document Format (PDF) will represent a published work according to the International Commission on Zoological Nomenclature (ICZN), and hence the new names contained in the electronic version are effectively published under that Code from the electronic edition alone. This published work and the nomenclatural acts it contains have been registered in ZooBank, the online registration system for the ICZN. The ZooBank LSIDs (Life Science Identifiers) can be resolved and the associated information viewed through any standard web browser by appending the LSID to the prefix http://zoobank.org/. The species registration is lsid:zoobank.org:act:BAC33FE2-1AF1-4801-A0EB-43EDD6F36DAB The LSID for this publication is: zoobank.org:pub:3031B111-771C-450F-B074-9795B709F9B2. The online version of this work is archived and available from the following digital repositories: PeerJ, PubMed Central and CLOCKSS.

### DNA extraction and molecular analyses

DNA was successfully extracted from 28 individuals collected at 10 stations during both KuramBio expeditions to the Kuril-Kamchatka Trench and adjacent abyssal plain (Fig. 1, Table 1).

**Table 1:** Stations where individuals of the species new to science was collected.

Expedition	Station code	Date	Position start		Position end		Depth (m)	No of ind.
			Latitude	Longitude	Latitude	Longitude		
KBI	3-9	2012-08-05	47°14.66′N	154°42.88′E	47°14.76′N	154°43.03′E	4,987–4,991	6
KBI	12-4	2012-09-01	39°42.78°N	147°09.55′E	39°42.49′N	147°09.37′E	5,224–5,215	1
KBII	17	2016-08-22	45°52.04′N	153°51.39′E	45°51.40′N	153°50.41′E	8,185.7–8,183.7	2
KBII	19	2016-08-23	45°52.02′N	153°51.15′E	45°51.41′N	153°50.21′E	8,192.7–8,187	2
KBII	28	2016-08-25	45°54.43′N	152°47.02′E	45°54.52′N	152°47.20′E	6,050.2–6,047.1	2
KBII	30	2016-08-27	45°56.38′N	152°56.70′E	45°56.83′N	152°50.93′E	6,228.3–6,163.7	3
KBII	40	2016-08-29	45°38.00′N	152°55.95′E	45°40.83′N	152°57.68′E	7,300.3–7,055.2	9
KBII	42	2016-08-30	45°39.62′N	152°56.39′E	45°40.26′N	152°57.63′E	7,110.6–7,119.6	1
KBII	85	2016-09-15	45°02.26′N	151°02.14′E	45°01.64′N	151°03.68′E	4,903.4–5,265.6	1
KBII	97	2016-09-18	44°05.68′N	151°24.88′E	44°06.94′N	151°24.88′E	6,440.4–6,560.7	1

**Notes.**

KBI KuramBio I KBII KuramBio II

In the case of specimens collected during KuramBio I expedition (two stations, seven individuals) a standard phenol-chloroform method following Hillis, Mable & Moritz (1996) was used. Air-dried DNA pellets were eluted in 100 µl of TE buffer, pH 8.00, stored at 4 °C until amplification, and subsequently at −20 °C long-term storage. The DNA extraction from the individuals collected during KuramBio II expedition (eight stations, 21 individuals) was performed on board with the use of 100 µl InstaGene™ Matrix (BIO-RAD, Hercules, CA, USA). The digestion step was carried out at 56 °C for 40 min. A fragment of Cytochrome Oxidase subunit I gene (COI; ca. 658 bp fragment) was amplified using standard LCO1490/HCO2198 (Folmer et al., 1994) or degenerated LCO1490-JJ/HCO2198-JJ (Astrin & Stüben, 2008) primers with DreamTaq Green PCR Mastermix (Thermo Fisher Scientific, Waltham, MA, USA) and reaction conditions following Hou, Fu & Li (2007). Sequences were obtained using BigDye sequencing protocol (3730xl; Applied Biosystems, Foster City, CA, USA) by Macrogen Inc., Korea (sequencing was performed either one or two ways). Sequences were edited using Geneious 10.1.2 leading to 28 sequences of 606–657 bp (excluding primers). In order to assess the number of MOTUs that could represent putative cryptic species the COI sequences were subjected to Barcode Index Number (BIN) System (Ratnasingham & Hebert, 2013) in Barcoding of Life Data Systems (BOLD). It compares newly submitted sequences with the sequences already available in BOLD. They are clustered according to their molecular divergence using algorithms aiming at finding discontinuities between clusters. Each cluster receives an unique and specific code (Barcode Index Number or BIN), either already available or new if submitted sequences do not cluster with already known BINs. Four chosen individuals representing three different BINs were taken for 16S gene analysis. It was amplified using the primer pair 16SFt_amp (GCRGTATIYTRACYGTGCTAAGG) and 16SRt_amp2 (CTGGCTTAAACCGRTYTGAACTC) (Lörz et al., 2018). PCR cycling conditions were as follows: initial denaturation in 95 °C for 2 min, followed by 35 cycles of 30 s at 95 °C, 30 s at 50°C, 45 s at 72 °C and final elongation in 72 °C for 5 min. Sequencing and editing was performed in the same way as for COI gene resulting in four sequences of 397–426 bp (excluding primers). All sequences were deposited in GenBank with the accession numbers for the 16S sequence: MH272096, MH272097, MH272098 and MH272099, and for the COI sequence: MH272100–MH272127 (Table S1). Relevant voucher information, taxonomic classifications, and sequences are accessible through the public data set “DS-RHACSAS” in BOLD (http://www.boldsystems.org) (Ratnasingham & Hebert, 2007).

Within the new species all COI sequences were aligned with MAFFT v7.308 algorithm (Katoh, Misawa & Kuma, 2002; Katoh & Standley, 2013) in Geneious 10.1.2 resulting in 606 bp alignment used to check the genetic relationships. The uncorrected p-distance and the Kimura 2-parameter (K2P) model (Kimura, 1980) were used to determine sequence divergence in MEGA V7.0.18 (Kumar, Stecher & Tamura, 2016). All obtained haplotypes were used to build Neighbour-Joining (NJ) tree based on the Kimura 2-parameter (Saitou & Nei, 1987). Node support was inferred with a bootstrap analysis (1,000 replicates) (Felsenstein, 1985). The published COI sequence of the genetically closest species of *Rhachotropis* (*R.* cf. *proxima,* GenBank accession number MG521128, Lörz et al., 2018) was used as outgroup (Table S1). To visualize molecular divergence of COI haplotypes, a Minimum Spanning Network was generated using PopART 1.7 (Bandelt, Forster & Röhl, 1999). To test whether the molecular distances between localities are correlated with the geographical proximity of sampled locations, Mantel tests of isolation by distance were performed as implemented in the program *Alleles in Space* (Miller, 2005). The level of correlation was assessed in SPSS 20 with Spearman’s rank correlation coefficient, and its statistical significance was tested with 1,000 bootstrap replicates. The test was performed on the basis of all samples but also excluding the most distant station (12-4) as only a single individual was collected there. Sequences of 16S gene were aligned similarly as the COI ones leading to 399 bp alignment. The 16S sequence of the same individual of *R.* cf. *proxima* as for COI analysis (Lörz, Jażdżewska & Brandt, 2018) was used as outgroup.

## Results

### Systematics

**Table:** 

**Figure 2:**
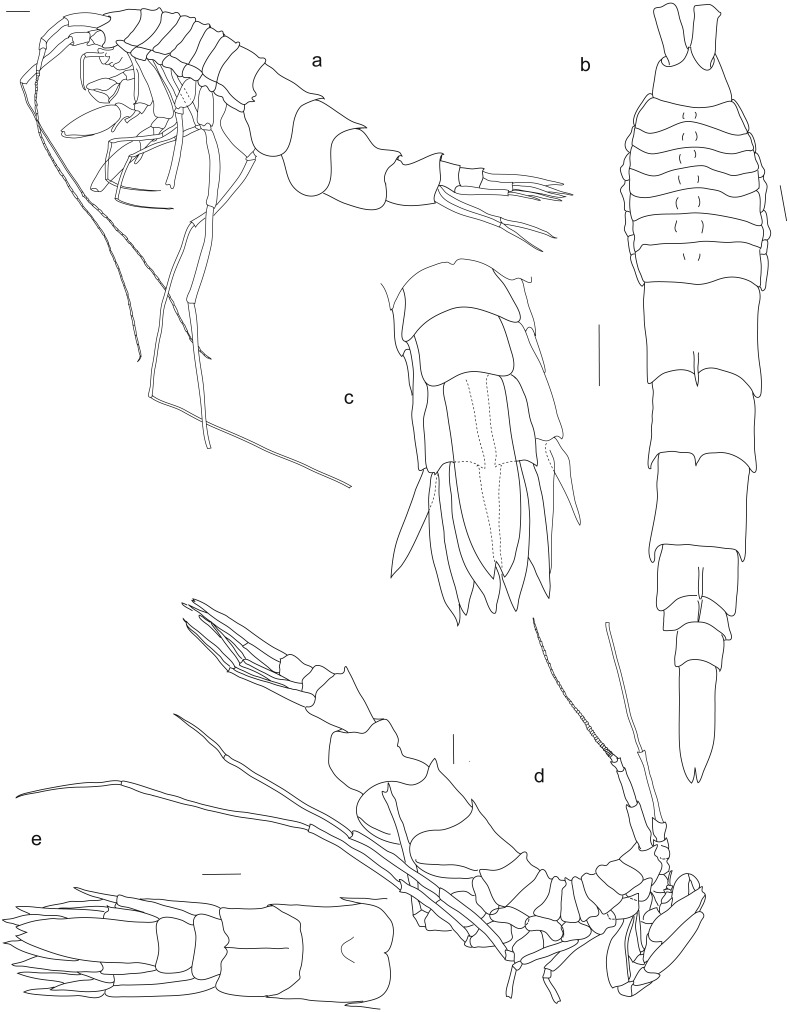
*Rhachotropis saskia* n. sp. Paratype SMF 51050, male, 23,8 mm, (A) lateral view, (B) dorsal view, (C) urosome dorsal; Holotype SMF 51046, female, 20.2 mm, (D) lateral view, (E) urosome dorsal. Scale bars: A–E 1 mm.

**Figure 3:**
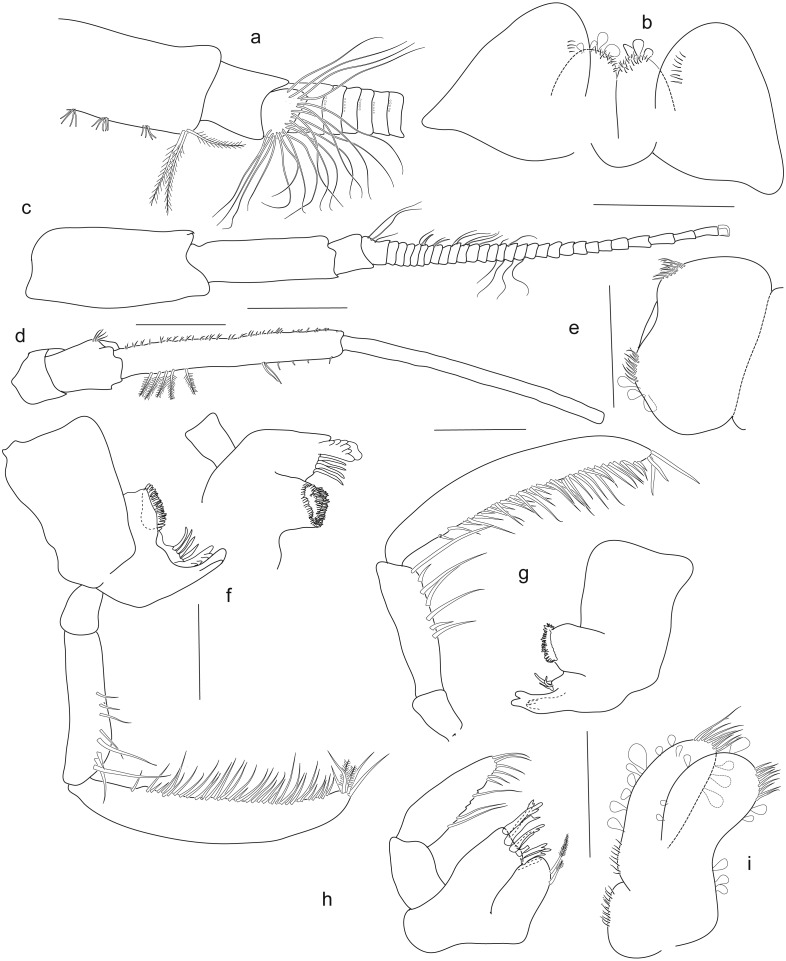
*Rhachotropis saskia* n. sp. Holotype SMF 51046, female 20.2 mm, (A) accessory flagellum, zoomed in and turned from C, (B) lower lip, (C) antenna 1, (D) antenna 2, (E) labrum, (F) left mandible, (G) right mandible, (H) maxilla 1, (I) maxilla 2. Scale bars: C, D 1 mm, B, E–I 0.5 mm.

**Figure 4:**
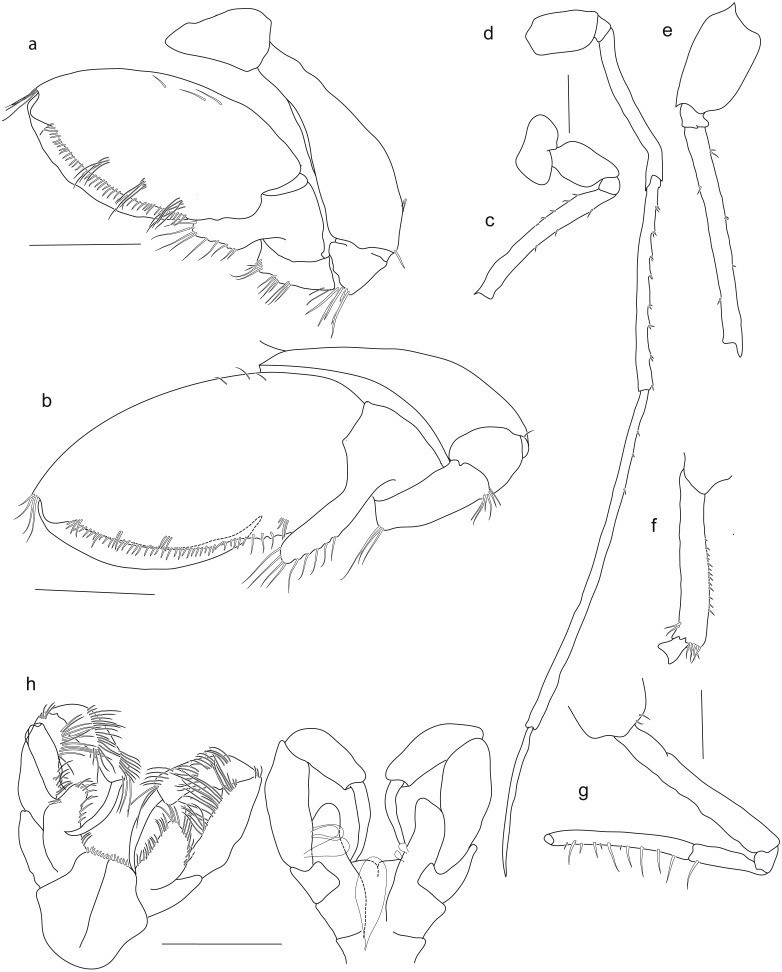
*Rhachotropis saskia* n. sp. Holotype SMF 51046, female 20.2 mm, (A) gnathopod1, (B) gnathopod 2, (C) pereopod 5, (D) pereopod 6, (E) pereopod 7, (F) pereopod 4, (G) pereopod 3, (H) maxilliped. Scale bars: A–E 1 mm.

**Figure 5:**
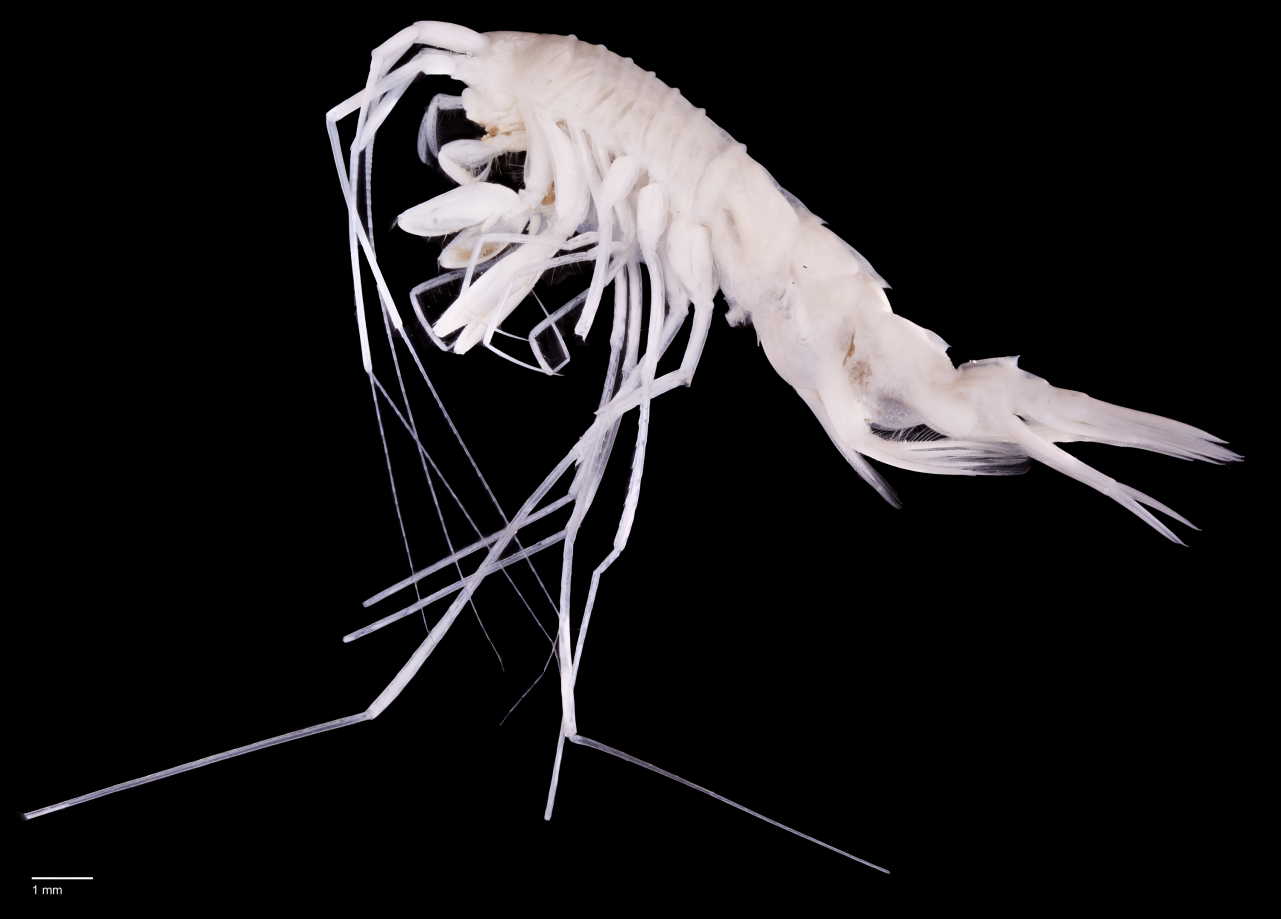
*Rhachotropis saskia* n. sp. Paratype SMF 51050, male, 23,8 mm, photographed after preservation. Scale bar 1 mm.

**Figure 6:**
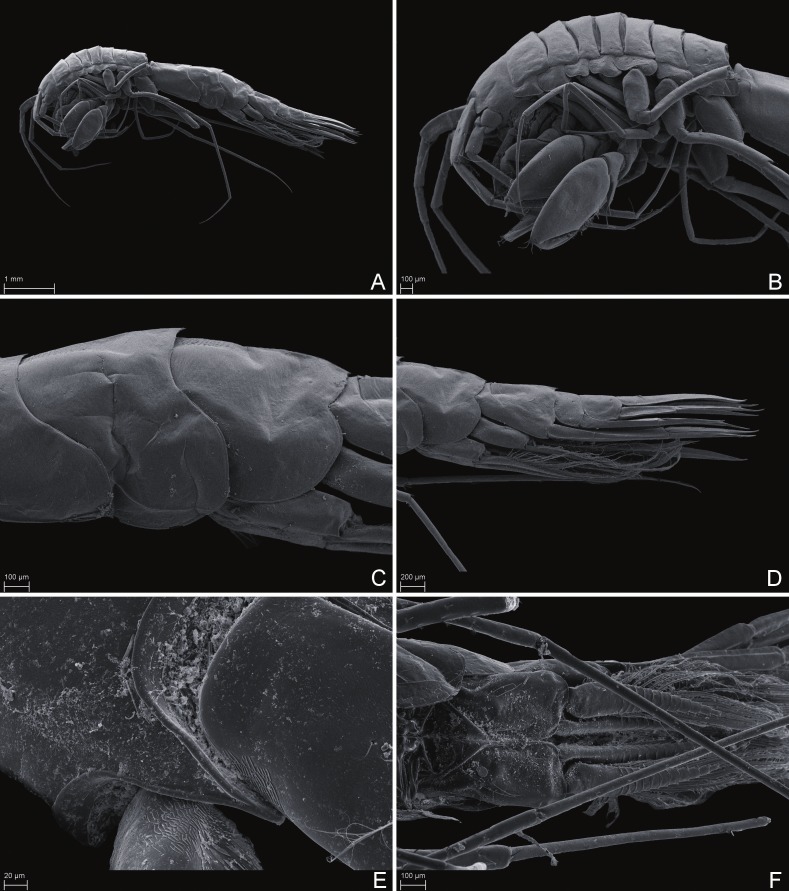
Scanning electron microscope images** of *Rhachotropis saskia* n. sp. SMF 51047, 6051 m; (A) habitus lateral, (B) head, pereon and gnathopods, (C) epimeral plates, (D) urosome, (E) rostrum, (F) pleopods from ventral.

**Figure 7:**
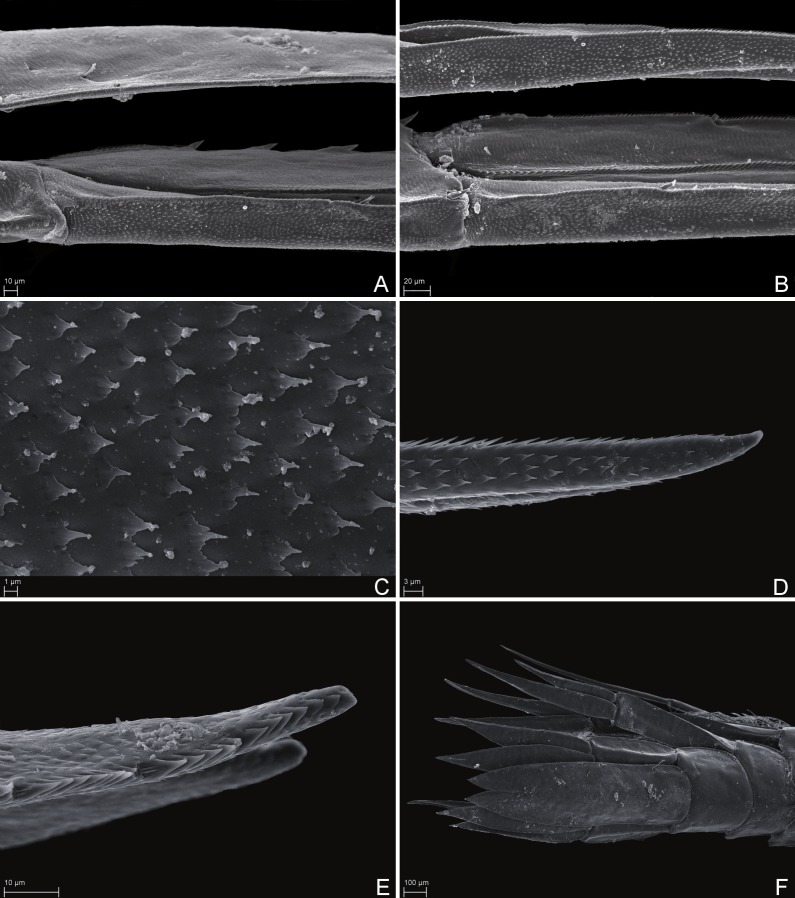
Scanning electron microscope images** of *Rhachotropis saskia* n. sp. SMF 51047, 6051 m; (A) telson and uropod 3 lateral view, (B) uropod 2 outer rami and uropod 1 inner rami lateral view, (C) detail of outer surface uropod 1 outer rami, (D) tip of outer rami uropod 3, (E) tip of rami uropod 1, (F) telson dorsal view.

**Figure 8:**
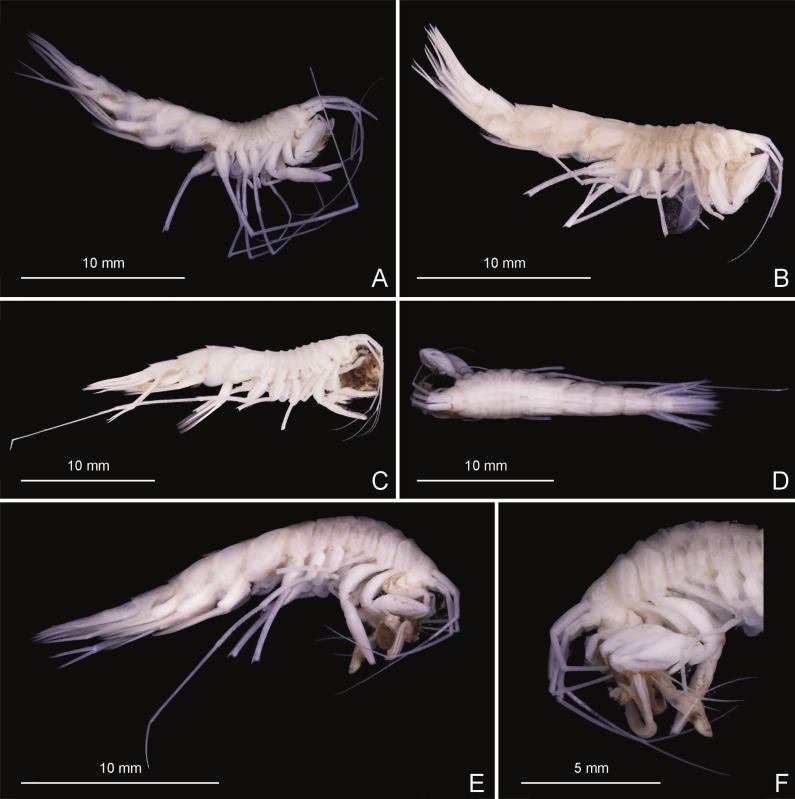
Stacked photographs of *Rhachotropis saskia* n. sp. (A) SMF 51049, male, 6,181 m, (B) SMF 51057, female, 7,081 m, (C) SMF 51045, male, 8,091 m lateral view, (D) SMF 51045, male, 8,091 m, dorsal view, (E) SMF 51061, male, 7,081 m, feeding most probably on polychaete, (F) SMF 51061, male, 7,081 m, same animal as shown in D from left view, feeding most probably on polychaete.

Order AMPHIPODA Latreille, 1816
Suborder GAMMARIDEA Latreille, 1802
Family EUSIRIDAE Stebbing, 1888
Genus *Rhachotropis* S.I. Smith, 1883
*Rhachotropis* S.I. Smith, 1883: 222.
*Gracilipes* Holmes, 1908: 526.
Type species *Oniscus aculeata* Lepechin, 1780
*Rhachotropis saskia* n. sp.
Lörz & Jażdżewska
(Figs. 2–8)

**Material examined**. Holotype: SMF 51046, female 20.2 mm, KuramBio II expedition 2016, station 19, gear EBS, sampling date 23.08.2016, depth 8,196 m depth, 45°52.02′N 153°51.15′E 45°51.41′N 153°50.21′.

Paratype: SMF 51050, male 23.8 mm; EBS 30, 27.08.2016, 6,181 m depth, 45°56.38′N 152°56.70′E 45°56.83′N 152°50.93′E.

Further material examined: SMF 51045, SMF 55047–55049, SMF 55051–55064 (detailed station data in the Table S1).

**Etymology**: This species is named after Saskia Brix. We highly acknowledge her enthusiasm for deep-sea research and greatly appreciate her outstanding contribution to peracarid taxonomy and systematics.

**Description**, based on holotype

No eyes. Rostrum short, slightly curved. Head dorsally smooth, all pereonites possess dorsal protuberances; pleonites 1–3 with dorsal spine, pointed posteriorly; epimeral plates ventrally rounded and posteriorly smooth. Urosome segment 1 with carina and dorsal spine, urosome 2 and 3 smooth.

*Antennae.* Antennae very long, antenna 1 slightly shorter than antenna 2, antenna 2 almost as long as body. Antenna 1 peduncle article 2 slimmer and slightly shorter than article 1, more than 2 times as long as article 3; flagellum 47-articulate; accessory flagellum 1-articulate. Antenna 2 peduncle article 3 and 4 not subequal in length, several plumose setae on fourth article; article 5 long, 1,8 length of article 4; flagellum 33-articulate.

*Mouthparts.* Mandible with incisor process well-developed; *lacinia mobilis* denticulate; molar process conical, large molar, edge with row of strong spines; palp article 1 short, about one-third length of article 2, article 3 twice as long as article 2, articles 2 and 3 with long slender setae, plumose setae at apical tip of left mandible palp. Maxilla 1 inner plate bearing 2 subterminal plumose setae; outer plate with 9 denticulate spines; article 1 of palp half the length of second article, article 2 of palp with several slender setae. Maxilla 2 inner and outer plates subequal in length, margins bearing stout and slender setae; inner plate slightly wider than outer plate. Maxilliped inner plate distally with short, thick spines; outer plate two times as long as inner plate, reaching half of article 2 of maxillipedal palp, outer plate margins strongly setose; palp article strongly setose. Labrum circular in shape, two setose areas anteriorly. Lower lip setose distoanteriorly, outer lobes separated by broad gap.

*Pereopods.* Coxa 1 anteriorly slightly drawn out. Gnathopods similar in shape, subchelate. Gnathopod 1 slightly smaller than gnathopod 2; basis bearing small spines at anteroventral corner; ischium and merus with long setae at posteroventral corner; carpus lobe extending width of propodus, spines at terminal end of lobe and along the posterior margin; propodus widened, oval; dactylus slender, reaching end of palm. Coxa 2 subquadrate; gnathopod 2 basis 1.5 times as wide as basis of gnathopod 1; ischium to dactylus similar to gnathopod 1.

Coxae 3 and 4 subquadrate. Pereopods 3 and 4 articles long and narrow; carpus, propodus and dactylus about same length.

Coxa 5 lobate. Pereopod 5 slender, as long as pereon; basis rectangular. Coxa 6 lobate, posterior lobe extending further ventrally than anterior lobe. Pereopod 6 larger, but similar in shape to pereopod 5. Coxa 7 posterior margin strongly drawn out. Pereopod 7 longer than pereopod 5 and 6, as long as body; basis with rounded extension posteriorly; merus posteroventral angle produced.

Pleopod 2 (right side) rami with 12 and 13 articles; rami same length, as long as peduncle.

Uropod 1 outer rami slightly shorter than inner rami; rami shorter than peduncle. Uropod 2 outer rami 0.3 times shorter than inner rami; peduncle shorter than outer rami and longer than inner rami. Uropod 3 inner rami slightly longer than outer rami, inner rami nearly twice as long as peduncle. Telson more than three times as long as wide, V-shaped insertion, cleft 10%.

**Epibionts** are dominant features of the mouthparts.

The new species is relatively easy to recognize due to prominent dorsal protuberances on all pereonites. It can be separated from other species of *Rhachotropis* by the combination of following characters: short rostrum; pereopod 7 longer than body; basis pereopod 7 linear; epimeral plate 3 entire posterior margin smooth; telson split one tenth.

### Molecular study

**Figure 9:**
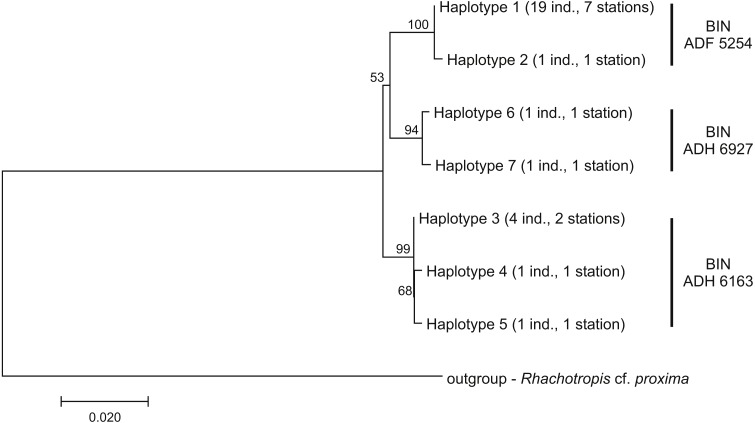
Neighbour-joining tree of the identified COI haplotypes with indication of the Barcode Identity Numbers (BINs) ascribed by Barcoding of Life Data Systems. The evolutionary distances were computed with Kimura 2-parameter method. The numbers in front of the nodes indicate bootstrap support (1,000 replicates, only the values higher than 50% are presented).

Among the 28 COI sequences, seven haplotypes forming three clusters were distinguished. These groups were associated with three different Barcode Identity Numbers (BINs) ascribed by BOLD (Fig. 9, Table S1). The overall mean sequence divergence expressed by both uncorrected p-distance and K2P was 0.009, while the divergence of the sequences within each cluster was variable and low (ranging from 0.0002 within ADF 5254 to 0.003 in ADH 6927) (Table 2). The distances between each pair of recognized BINs was 0.019. The value was the same for both uncorrected *p*-distance and K2P.

**Table 2:** Overall genetic distance (*p*-distance and K2p) of COI sequences and the mean distances within the ascribed BINs.

	No of ind.	No of haplotypes	*p*-distance	K2p
Overall	28	7	0.009	0.009
ADF 5254	20	2	0.0002	0.0002
ADH 6163	6	3	0.001	0.001
ADH 6927	2	2	0.003	0.003

The mean genetic distance to the closest known congener (*R.* cf. *proxima*) was 0.175 and 0.200 of *p*-distance and K2P, respectively (Table 3).

**Table 3:** Genetic distances between the new species and outgroup (*Rhachotropis cf. proxima*) inferred from COI sequences.

	*p*-distance	K2p
Overall-outgroup	0.175	0.200
ADF 5254-outgroup	0.177	0.202
ADH 6163-outgroup	0.172	0.196
ADH 6927-outgroup	0.173	0.198

**Figure 10:**
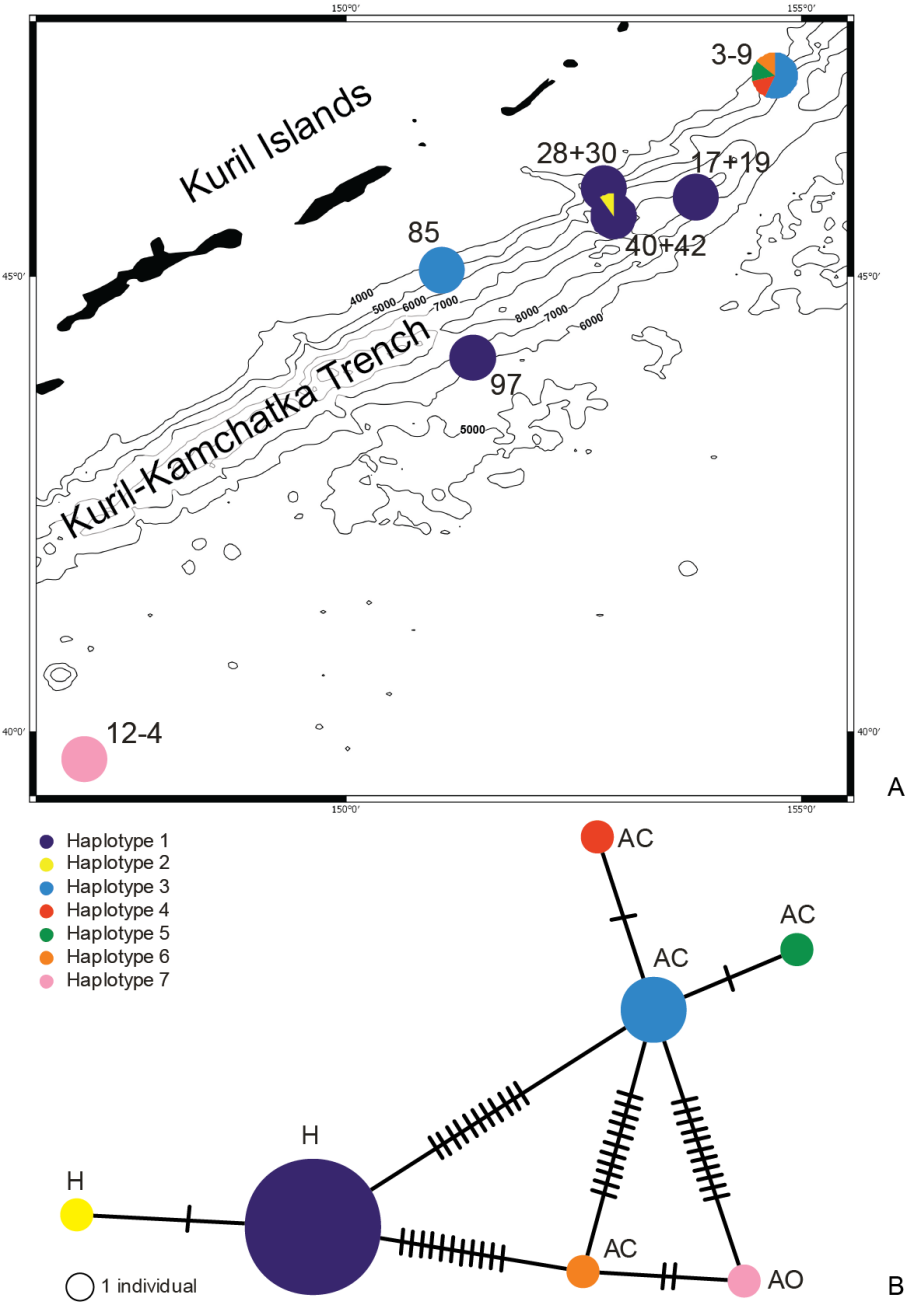
Distribution of the haplotypes (A) and Minimum Spanning Network (B). Each line represents a mutation between sequences. The size of the circles is proportional to the frequency of the haplotypes, location of the haplotype: H, hadal; AC, abyssal on the continental side of KKT; AO, abyssal on the ocean side of KKT.

The study of 16S gene resulted in two haplotypes differing in two positions and the mean intraspecific distance of 0.001 (both *p*-distance and K2P). The divergence between the sequences of the newly described species and *R.* cf. *proxima* was 0.165 (*p*-distance) and 0.186 (K2P).

**Figure 11:**
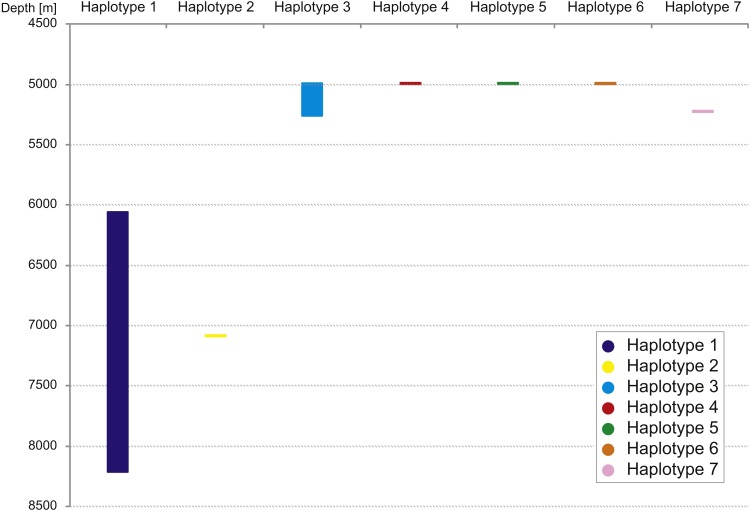
Depth distribution of COI haplotypes.

The haplotype distribution showed that there were no shared haplotypes between neither the stations situated on both sides of the trench nor between abyssal and hadal zones (Fig. 10). Only a single haplotype was found on the ocean side of KKT. This single haplotype was quite distant from all other ones, however, the haplotype that forms the same clade on the NJ tree (and differ only with two mutations) was found at the station on the other side of the trench 1,000 km apart. At the same time at the northernmost station (3–9) there co-existed haplotypes from two different NJ tree clades. The minimum spanning network indicated that the two haplotypes from deep-water stations (haplotypes 1 and 2) separated from all others. The bathymetric distribution of the haplotypes disclosed one that has a very wide depth range (haplotype 1, >2,000 m), while other ones were restricted to narrow and relatively shallow depths (Fig. 11). The abyssal is characterized by higher haplotype richness. The isolation by distance test indicated that there exists a correlation between the genetic and geographical distances regardless of the dataset used (Spearman rank order correlation, *R* = 0.573, *P* = 0.001 when all samples were included, and *R* = 0.668, *P* = 0.001 for dataset without station 12-4).

## Discussion

### Morphological distinctions of *Rhachotropis saskia* n. sp.

The only benthic *Rhachotropis* from hadal depth determined to species level so far is *R*. *flemmingi*, reported from the Sunda Trench and the Kuril-Kamchatka Trench (Dahl, 1959; Kamenskaya, 1997; Jamieson, 2015). The morphological characters separating the two hadal *Rhachotropis* species are listed in Table 4. Several *Rhachotropis* individuals have been noted from hadal depth in too poor condition to be described (Dahl, 1959; Lörz et al., 2012). Kamenskaya (1981) noted *Rhachotropis* sp. A from the KKT from 6,200–8,015 m and briefly described it but without any illustration. It differs from *R. saskia* n. sp. in the shape of the antenna. *Rhachotropis* sp. A has the 3rd article of the peduncle of antenna 1 longer than the 2nd and the flagellum is a little longer than peduncle, whereas in *R. saskia* n. sp. the flagellum is distinctly longer than the peduncle and the third article of the peduncle antenna 1 is only a fifth of the length of the second article.

**Table 4:** Morphological separation of hadal *Rhachotropis*.

	*Rhachotropis saskia* n. sp.	*R. flemmingi* Dahl, 1959
Size	18–23 mm	18 mm
Depth (m)	4,987–8,196	7,160
Rostrum	short	long
Basis pereopod 7	rounded distal corner	acute distal corner
Third epimeral plate	smooth	strongly serrate
Telson	cleft 10%	cleft 20%
Telson distal part	V- Shaped	tapering to point

The new species increases the known *Rhachotropis* from the North-West Pacific to seven species. Therefore, a key for determination via outer morphological structures is provided below.

**Key to the** ***Rhachotropis*** **from the NW Pacific**:

**Table:** 

1.	Eyes present	2
	Eyes absent	4
2.	Pereonites 6 and 7 bearing strong lateral protrusions	*R. aculeata* (Lepechin, 1780)
	Pereonites 6 and 7 laterally smooth	3
3.	Telson minutely cleft, coxa 1 slightly elongated	*R. natator* (Holmes, 1808)
	Telson cleft half its length, coxa 1 drawn out width of head	*R. macropus* Sars, 1893
4.	Head with strong dorsal protrusion	*R. marinae* Lörz, Jażdżewska & Brandt, 2018
	Head dorsally smooth	5
5.	Rostrum extending beyond acute anterior head lobe	6
	Rostrum short, third epimeral plate smooth	*R. saskia* n. sp.
6.	Third epimeral plate strongly serrate, telson incised 20%	*R. flemmingi* Dahl, 1959
	Third epimeral plate weakly serrate, telson cleft 10%	*R. distincta* (Holmes, 1908)

### Ecological features, locomotion and feeding

Two individuals of *Rhachotropis saskia* n. sp. were encountered holding Polychaeta in their gnathopods and mouthparts (see Figs. 8E, 8F). The non-photographed Polychaeta was identified on board as *Ophelina* sp. (Opheliniidae, Polycheta) (I Alalykina, pers. comm., 2016). The generic characters of *Rhachotropis* mouthparts: strongly spinose mandibles with strongly dentate molar, the maxilla plate possessing several terminal spines, and very long maxilliped, clearly visible from lateral view, enable capture and fragmenting of their prey. Clearly *Rhachotropis saskia* n. sp. is the predator and the worm its prey. Kamenskaya (1981) and Kamenskaya (1984) studied the mouthparts morphology and digestive tract of deep-sea amphipods and confirmed the predatory habit of species from genera *Eusirus* and *Rhachotropis*. Pirlot (1936) reported that among the deep-sea species of the Dutch ‘Siboga’ expedition (1899–1900) there was a group of amphipods thought to occur directly on the sediment surface. He reported that the most striking characteristic of this group, represented by *Lepechinella curvispinosa* Pirlot, 1933 (Lepechinellidae) and *Rhachotropis siboge* Pirlot, 1934 (Eusiridae), was their long and slender bodies, often with dorsal projections with very long and slender appendages comparable to other amphipods. With a polychaete being eaten caught *in situ* we must assume that *Rhachotropis saskia* n. sp. can hunt swimming prey such as amphipods as well as supra- or epibenthic animals such as polychaetes while walking on the soft sediment—as suggested by Pirlot (1936) and being observed for *Princaxelia* (Jamieson et al., 2012). The soft sediment of the KKT where the present material was caught supports this idea.

*Rhachotropis*—as all amphipods—are brooders, without free-living larval stage, so their expansion depends on the dispersal abilities of juveniles and adults. The body shape of representatives of this genus is ideal for fast swimming. Boudrias (2002) studied the functional morphology of pleopods of the deep-sea scavenger *Eurythenes gryllus* and summarised the complex skeleton-musculature construction. The ancillary structures of the propulsive limbs interact strongly with the fluid dynamics forces affecting their locomotion. On first view the pleopods of *Rhachotropis* in general seem very similar to those of *E. gryllus.* To live in a trench with rare food availability requires good senses when food is available and fast swimming abilities for carnivores to get there to either feed of a large item or—more likely—feed on the feeding scavengers (e.g., lysianassid amphipods). Jamieson et al. (2012) measured *in situ* locomotion and feeding behaviour of *Princaxelia* species from hadal depths. These amphipods have the capacity for long range swimming, high manoeuvrability in close range, and efficient predatory behaviour, these criteria also match *Rhachotropis*. However, *Princaxelia* is a much more robust amphipod than *Rhachotropis.* Both genera group fast swimming predators, but it seems more imaginable that *Princaxelia* actively hunts swimming, solid scavengers (such as lysianassoids) and *Rhachotropis* stalks over the seabed on its delicate legs hunting polychaetes.

Different deep-sea Amphipoda, e.g., *Bathycallisoma schellenbergi* (Birstein & Vinogradov, 1958), *Princaxelia abyssalis* (Dahl, 1959) are known to occur in several trenches (Kamenskaya, 1997; Lörz, 2010; Lacey et al., 2017) and those crustaceans are known to be good swimmers. The complex hydrography of the KKT area with currents flowing in different directions and local eddies near the Bussol Strait (Arseniev & Leontieva, 1970; Uehara & Miyake, 1999; Rabinovich, Thomson & Bograd, 2002; Kawabe & Fujio, 2010) could also potentially facilitate migrations of the epibenthic Amphipoda such as *Rhachotropis saskia* n. sp.

### Molecular diversity, horizontal and vertical distribution pattern

Our study revealed three lineages within the studied species with different BINs ascribed by BOLD. The haplotypes within each BIN were almost identical (less than 0.003 of uncorrected *p*-distance and K2P) while the differences between lineages reached 0.019 of both studied distance values. The overall sequence divergence of the studied species (Table 2) remained within the threshold limit set at the level of 0.03 which is commonly used for crustacean species delineation and amphipods in particular (e.g., Hebert, Ratnasingham & DeWaard, 2003; Costa et al., 2007; Costa et al., 2009; Raupach et al., 2015; Lobo et al., 2017). Some authors noticed that for deep-sea Amphipoda the barcoding gap can be observed even at higher level—between 6 and 12% of COI sequence divergence (Knox et al., 2012). In other studies of the genus *Rhachotropis* the value of mean intraspecific COI sequence diversity was variable, ranging from 0.000 to 0.058 (Lörz, Jażdżewska & Brandt, 2018; Lörz et al., 2018). The barcoding of five individuals of recently described *Rhachotropis marinae* Lörz, Jażdżewska & Brandt, 2018 from the abyssal of the Sea Of Okhotsk disclosed five haplotypes of mean divergence 0.014 (both studied values) (Lörz, Jażdżewska & Brandt, 2018). It is important to note that in the cited case all haplotypes came from a single station. The 16S gene sequencing of representatives of each clade also confirmed that the presently described species represent a single genetic unit as there was no significant differences between the haplotypes. It is worth noting that no morphological variation is associated with the three lineages recognized on the basis of COI gene. It seems that ascribing the three BINs by BOLD was over splitting of the single taxonomical unit. In such cases the use of additional genes (such as slower evolving 16S) may help in correct species delimitation.

The most distant stations where *R. saskia* was collected (St. 3-9 and 12-4) are 1,000 km apart and are separated by Kuril-Kamchatka Trench but the signal of isolation by distance was recorded. Kobayashi et al. (in press) who studied the distribution of the polychaete *Sternaspis* cf. *williamsae* Salazar-Vallejo & Buzhinskaja, 2013 in the abyssal area along KKT and east of Japan found no genetic structure associated with geographic distance for this species. Also, the existence of the Mid-Atlantic Ridge did not act as a barrier for gene flow as it was proved for different mollusc species (Zardus et al., 2006; Etter et al., 2011). The lack of isolation by distance in the above mentioned groups can be associated with their biology. In the ontogenesis they go through pelagic larval stage that can be easily moved with bottom currents and colonize new areas. Amphipods belong to brooding species, that do not possess a free-living larval stage so their dispersal abilities and resulting gene flow seem to be restricted, especially if they present benthic lifestyle. A comparison of the presently obtained data with the existing literature concerning abyssal or hadal Amphipoda is difficult because there are not many genetic studies conducted at similar spatial scales for other species. The available publications usually study widely distributed taxa and compare single samples collected in very remote areas (e.g., *Ventiella sulfuris* Barnard & Ingram, 1990 in France, Hessler & Vrijenhoek, 1992, *Eurythenes gryllus* (Lichtenstein in Mandt, 1822) s.l. in (Havermans et al., 2013) or *Paralicella* spp. in Ritchie, Jamieson & Piertney, 2017). The literature dealing with another peracarid brooder group, the Isopoda, brings ambiguous results. The lack of isolation by distance and no influence of submarine physiographic barrier on the population structure was observed for *Haploniscus rostratus* (Menzies, 1962) in the South Atlantic (Brix, Riehl & Leese, 2011). These authors found only a single COI haplotype among the individuals collected on both sides of the Walvis Ridge. Moderate influence of the Mid-Atlantic Ridge on the population structure of some macrostylids isopods was also recorded by Riehl, Lins & Brandt (2018). To explain this phenomenon passive transport with bottom currents of these animals has been proposed Riehl, Lins & Brandt (2018).

However, another study of the isopods of the central Atlantic revealed that the Mid-Atlantic Ridge constitutes the barrier for gene flow in case of certain families, while for other ones it is not a limiting factor (Bober et al., 2018). The authors concluded that the swimming abilities of representatives of particular families are crucial for the gene flow over the studied barrier. In the case of presently studied species the passive transport associated with different currents recognized in the studied area may play the role for the wider distribution of the species. The northward current originating from the Antarctic Bottom Water and floating into North Pacific was argued as the reason for the affinities between the genera of Caprellidea recorded in the Southern Ocean and in the Japan Trench (Takeuchi, Tomikawa & Lindsay, 2016). This current can be responsible for *R. saskia* haplotypes distribution. One has to take into account, however, that the hydrography of the KKT area is as well under the influence of local currents, including the eddy in the Bussol’ Strait region (Rabinovich, Thomson & Bograd, 2002) that may influence the ranges of abyssal and hadal lineages. Study of more individuals collected along the long axis of the trench as well as across it may help answering this question. Active movement as a driving force for population mixing can also be expected since the genus *Rhachotropis* groups species of good swimming abilities with some species regarded as truly pelagic (Birstein & Vinogradov, 1955). The body shape of the herein described species is ideal for swimming, as it possesses strong pleopods that help it in active movement proving its migration possibilities over the bottom. One has to take into account, however, that isolation due to distance was recorded for this species so the mixing of its populations does not have to be constant.

**Table 5:** Benthic Amphipoda sampled in the Kuril-Kamchatka Trench area below 2,000 m, modified from Kamenskaya (1981) and Kamenskaya (1997).

Taxon	Author	Depth range world ocean (m)	Depth range KKT (m)	Feeding type
*Ampelisca unsocalae*	Barnard (1960)	400–4,927	1,610–4,927	F
*Neohela pacifica*	Gurjanova (1953)	<2,550	2,550–2,630	D
*Uschakoviella echinophora*	Gurjanova (1955)	54–2,630	2,550–2,630	D
*Rhachotropis aculeata*	Lepechin (1780)	20–2,630	2,550–2,630	C
*Aceroides* sp.		–	2,550–2,630	D
*Lepechinella uchu*	Barnard (1973)	2,770–3,563	2,770–2,820	F
*Lepechinella arctica*	Schellenberg (1926)	600–2,820	2,770–2,820	F
*Neohela* sp.^a^		–	2,960	D
*Harpinia* sp. A		–	2,960	C
*Leucothoe* sp.		–	4,560	Co
*Rhachotropis saskia* n. sp.	Lörz & Jażdżewska (2018)	–	4,990–8,196	C
*Amathillopsis* sp.		5,045–5,005	5,045–5,005	D
*Byblisoides arcillis*	Barnard (1961)	2,000–6,571	5,045–5,005	F
*Aberratylus aberrantis*	Barnard (1962)	788–6,330	5,200	F
*Epimeria abyssalis*	Shimomura & Tomikawa (2016)	5,475–5,695	4,575–5,695	?
*Rhachotropis flemmingi*	Dahl (1959)	6,090–7,160	6,090–6,135	C
*Rhachotropis* sp. A^a^		6,200–9,750	6,200–8,015	C
*Bathycallisoma schellenbergi*	Birstein & Vinogradov (1958)	5,560–9,875	6,435–8,000	C
*Princaxelia abyssalis*	Dahl (1959)	6,435–9,530	6,435–9,345	C
*Metaceradocoides vitjazi*	Birstein & Vinogradova (1960)	6,835–8,900	6,835–8,345	D
*Epimeria* sp.^a^		–	7,180	D
*Harpinia* sp. B		–	7,180	C
*Astyroides carinatus*	Birstein & Vinogradova (1960)	7,210–7,230	7,2100-7,230	?
*Lepechinella ultraabyssalis*	Birstein & Vinogradova (1960)	6,475–8,135	7,370–8,135	F
*Phippsiella* sp. B		–	7,550–7,600	C
*Stegocephelus* sp.		–	7,820–8,040	C
*Hirondellea gigas*	Birstein & Vinogradov (1955)	6,000–1,0920	8,035–9,345	C

**Notes.**

a Indicates species considered as new to science by Kamenskaya (1997) but never described. Feeding types (following Kamenskaya, 1997).

C carnivorous (including scavengers) D detritiphagous F filter-feeders/suspension-feeders Co commensal

**Figure 12:**
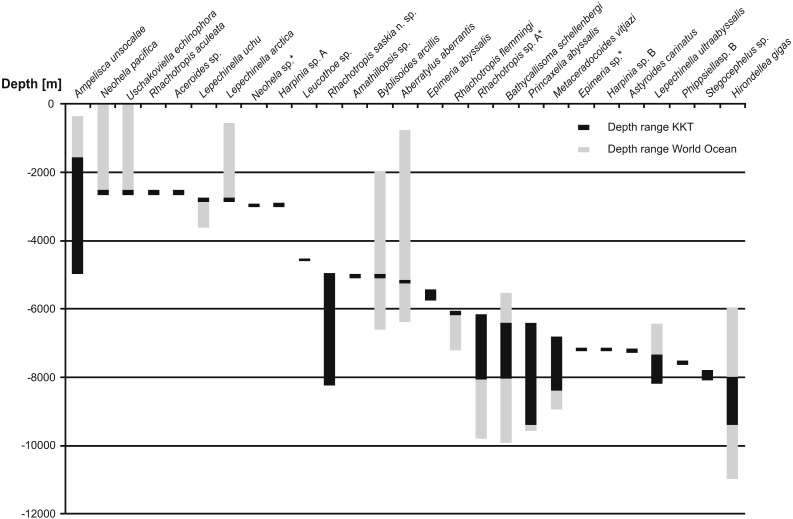
Depth ranges of benthic species recorded in Kuril Kamchatka Trench area. Only the species recorded in KKT below 2,000 m are shown. The species are organized by the shallowest record in KKT. Grey bars indicate the known depth range, the black bars show the depth range in KKT area.

So far 27 benthic species (including the ones identified as separate species but not described yet) were reported from the KKT below 2,500 m, 13 of these are known from depths below 6,000 m (Table 5, Fig. 12) (Kamenskaya, 1997; Shimomura & Tomikawa, 2016). Among them *R. saskia* n. sp. appeared to have very wide bathymetric range (>3,000 m) occurring both in abyssal and hadal zones of the studied area. There are only seven deep-sea amphipod species recorded from this area with such a large depth distribution. However, the ranges of three of them (*Ampelisca unsocalae* Barnard, 1960*, Byblisoides arcillis* Barnard, 1961*, Aberratylus aberrantis* (Barnard, 1962)) seem doubtful as according to the classification of oceanic zones they inhabit as many as three of them –from shelf to abyss in case of the first species and from bathyal to hadal depths for the remaining two. Future molecular investigations might prove the morphological determination of some of these species’ specimens as wrong. Recently a new amphipod species having wide bathymetric distribution (2,000 m) and inhabiting both abyssal and hadal zones was described from Japan Trench. The species was collected in the cold seep areas and, according to the authors, it may be endemic for this type of habitat (Takeuchi, Tomikawa & Lindsay, 2016). This may explain its large bathymetric range since the special type of environment may be more important for it than the actual depth. A wide bathymetric range was reported for some *Rhachotropis* species (*R. natator* (Holmes, 1908) and *R. anoculata* Barnard, 1962) (Lörz et al., 2012); however, the above cited distribution records were based on morphological results only. The observed depth range of *R. saskia* n. sp. is a striking finding as in molecular studies concerning crustacean species, originally regarded as eurybathic, it appeared that many of these species are in fact cryptic or pseudo-cryptic species complexes which have much narrower depth ranges than anticipated (Havermans et al., 2013; Brix, Svavarsson & Leese, 2014). More differences in vertical rather than horizontal genetic separation of species was observed in several invertebrate groups (e.g., Zardus et al., 2006; Etter et al., 2011; Schüller, 2011; Lörz et al., 2012; Eustace et al., 2016). On the basis of above cited literature the phylogeographic barrier for gene flow of marine invertebrates was set at 3,000–3,500 m depth which coincides with the commonly accepted transition between bathyal and abyssal. The presence of an ecotone zone between abyssal and hadal was suggested to exist at the depths 6,000–7,000 m (Kamenskaya, 1981; Kamenskaya, 1995; Jamieson et al., 2011). The new Kuril-Kamchatka species does not only cross the transitional zone between abyssal and hadal depths, it even possesses a large hadal population. Thus, this species is adapted to a considerable range of hydrostatic pressure. It is already known that some deep-sea amphipod species may be resistant to such pressure changes (Havermans et al., 2013). Nevertheless, it is important to note that the individuals constituting the hadal population of *R. saskia* n. sp. are genetically separated from those inhabiting shallower waters and do not share haplotypes regardless of their relative geographic proximity at the studied stations. Future analyses of further material from additional stations and areas would allow to assess to what extent the hadal population is isolated from the abyssal one.

## Conclusion

A large sized amphipod of the genus *Rhachotropis* sampled in the Kuril-Kamchatka Trench is new to science and increases the number of described hadal species of that area to eight. This predatory species, *Rhachotropis saskia* n. sp., is sampled on the continental and ocean abyssal margins of the Kuril-Kamchatka Trench as well as at hadal depths of 8,196 m. The wide bathymetric distribution of the new species over more than 3,000 m is confirmed by molecular analysis, indicating that the Kuril Kamchatka Trench is not a biogeographic distribution barrier for this species.

##  Supplemental Information

10.7717/peerj.4887/supp-1 Table S1 New species accession numbers in BOLD, GenBank and station data. AMPIV001-17 - *Rhachotropis* cf. *proxima* used as an outgroup

10.7717/peerj.4887/supp-2 Supplemental Information 1 Fasta file 16 S

10.7717/peerj.4887/supp-3 Supplemental Information 2 Fasta file COI

## Additional Information and Declarations

### Field Study Permissions

The following information was supplied relating to field study approvals (i.e., approving body and any reference numbers): Sampling permission was provided via Russia port authority, permission 49 from 5.4.2016.

### DNA Deposition

The following information was supplied regarding the deposition of DNA sequences: All sequences were deposited in GenBank with the accession numbers for the 16S sequence: (MH272096, MH272097, MH272098 and MH272099) and the COI sequence (MH272100–MH272127) and are also available in the Supplemental Information. Relevant voucher information, taxonomic classifications, and sequences are accessible through the public data set ”DS-RHACSAS” in BOLD (http://www.boldsystems.org).

### Data Availability

The following information was supplied regarding data availability: The amphipod material is deposited in the Zoological Museum Frankfurt, Germany.

### New Species Registration

The following information was supplied regarding the registration of a newly described species: *Rhachotropis saskia* n.sp. Lörz & Jażdżewska - the manuscript registration: urn:lsid:zoobank.org:pub:3031B111-771C-450F-B074-9795B709F9B2 - the species registration: urn:lsid:zoobank.org:act:BAC33FE2-1AF1-4801-A0EB-43EDD6F36DAB.
