# Supplementary material for: A new predator connecting the abyssal with the hadal in the Kuril-Kamchatka Trench, NW Pacific
Source: PeerJ. 2018 Jun 7;6:e4887. doi: 10.7717/peerj.4887 (PMC5994337; doi:10.7717/peerj.4887)
Supplement: Table S1 [file peerj-06-4887-s001.pdf]

Supplementary table 1. New species accession numbers in BOLD, GenBank and station data. AMPIV001-17 - *Rhachotropis* cf. *proxima* used as an outgroup

| Process ID | Sample ID | Expedition | Station | Position start | | Position end | | Depth [m] | Date | ZMK ID | BIN | GenBank | |
|---|---|---|---|---|---|---|---|---|---|---|---|---|---|
| | | | | Latitude | Longitude | Latitude | Longitude | | | | | 16S | COI |
| AJAKK454-16 | 3-9S_Eusi4_2012_1 | KBI | 3-9 | 47° 14.66' N | 154° 42.88' E | 47° 14.76' N | 154° 43.03' E | 4987-4991 | 2012-08-05 | SMF 51051 | BOLD:ADH6163 | x | MH272122 |
| AJAKK455-16 | 3-9S_Eusi4_2012_2 | KBI | 3-9 | 47° 14.66' N | 154° 42.88' E | 47° 14.76' N | 154° 43.03' E | 4987-4991 | 2012-08-05 | SMF 51053 | BOLD:ADH6163 | x | MH272107 |
| AJAKK457-16 | 3-9S_Eusi4_2012_4 | KBI | 3-9 | 47° 14.66' N | 154° 42.88' E | 47° 14.76' N | 154° 43.03' E | 4987-4991 | 2012-08-05 | SMF 51054 | BOLD:ADH6163 | x | MH272111 |
| AJAKK459-16 | 3-9S_Eusi4_2012_6 | KBI | 3-9 | 47° 14.66' N | 154° 42.88' E | 47° 14.76' N | 154° 43.03' E | 4987-4991 | 2012-08-05 | SMF 51055 | BOLD:ADH6163 | x | MH272113 |
| AJAKK462-16 | 3-9S_Eusi4_2012_9 | KBI | 3-9 | 47° 14.66' N | 154° 42.88' E | 47° 14.76' N | 154° 43.03' E | 4987-4991 | 2012-08-05 | SMF 51056 | BOLD:ADH6927 | MH272097 | MH272118 |
| AJAKK463-16 | 3-9S_Eusi4_2012_10 | KBI | 3-9 | 47° 14.66' N | 154° 42.88' E | 47° 14.76' N | 154° 43.03' E | 4987-4991 | 2012-08-05 | SMF 51052 | BOLD:ADH6163 | x | MH272119 |
| AJAKK476-16 | 12-4S_Eusi4_2012_1 | KBI | 12-4 | 39° 42.78° N | 147° 09.55' E | 39° 42.49' N | 147° 09.37' E | 5224-5215 | 2012-09-01 | SMF 51045 | BOLD:ADH6927 | x | MH272117 |
| AJAKK988-17 | 17E_Rhac1_2016_1 | KBII | 17 | 45° 52.04' N | 153° 51.39' E | 45° 51.40' N | 153° 50.41' E | 8185.7-8183.7 | 2016-08-22 | SMF 51045 | BOLD:ADF5254 | x | MH272109 |
| AJAKK927-17 | 17S_Rhac_2016_1 | KBII | 17 | 45° 52.04' N | 153° 51.39' E | 45° 51.40' N | 153° 50.41' E | 8185.7-8183.7 | 2016-08-22 | x | BOLD:ADF5254 | x | MH272103 |
| AJAKK938-17 | 19S_Rhac_2016_1 | KBII | 19 | 45° 52.02' N | 153° 51.15' E | 45° 51.41' N | 153° 50.21' E | 8192.7-8187 | 2016-08-23 | x | BOLD:ADF5254 | x | MH272112 |
| AJAKK989-17 | 19S_Rhac1_2016_2 | KBII | 19 | 45° 52.02' N | 153° 51.15' E | 45° 51.41' N | 153° 50.21' E | 8192.7-8187 | 2016-08-23 | SMF 51046 | BOLD:ADF5254 | x | MH272127 |
| AJAKK990-17 | 28E_Rhac1_2016_1 | KBII | 28 | 45° 54.43' N | 152° 47.02' E | 45° 54.52' N | 152° 47.20' E | 6050.2-6047.1 | 2016-08-25 | SMF 51047 | BOLD:ADF5254 | MH272096 | MH272100 |
| AJAKK991-17 | 28S_Rhac1_2016_1 | KBII | 28 | 45° 54.43' N | 152° 47.02' E | 45° 54.52' N | 152° 47.20' E | 6050.2-6047.1 | 2016-08-25 | SMF 51048 | BOLD:ADF5254 | x | MH272121 |
| AJAKK934-17 | 30S_Rhac_2016_1 | KBII | 30 | 45° 56.38' N | 152° 56.70' E | 45° 56.83' N | 152° 50.93' E | 6228.3-6163.7 | 2016-08-27 | x | BOLD:ADF5254 | x | MH272126 |
| AJAKK992-17 | 30S_Rhac1_2016_2 | KBII | 30 | 45° 56.38' N | 152° 56.70' E | 45° 56.83' N | 152° 50.93' E | 6228.3-6163.7 | 2016-08-27 | SMF 51049 | BOLD:ADF5254 | x | MH272106 |
| AJAKK993-17 | 30S_Rhac1_2016_3 | KBII | 30 | 45° 56.38' N | 152° 56.70' E | 45° 56.83' N | 152° 50.93' E | 6228.3-6163.7 | 2016-08-27 | SMF 51050 | BOLD:ADF5254 | x | MH272116 |
| AJAKK945-17 | 40E_Rhac_2016_1 | KBII | 40 | 45° 38.00' N | 152° 55.95' E | 45° 40.83' N | 152° 57.68' E | 7300.3-7055.2 | 2016-08-29 | x | BOLD:ADF5254 | x | MH272104 |
| AJAKK996-17 | 40S_Rhac1_2016_2 | KBII | 40 | 45° 38.00' N | 152° 55.95' E | 45° 40.83' N | 152° 57.68' E | 7300.3-7055.2 | 2016-08-29 | SMF 51057 | BOLD:ADF5254 | x | MH272120 |
| AJAKK997-17 | 40S_Rhac1_2016_3 | KBII | 40 | 45° 38.00' N | 152° 55.95' E | 45° 40.83' N | 152° 57.68' E | 7300.3-7055.2 | 2016-08-29 | SMF 51058 | BOLD:ADF5254 | x | MH272102 |
| AJAKK998-17 | 40S_Rhac1_2016_4 | KBII | 40 | 45° 38.00' N | 152° 55.95' E | 45° 40.83' N | 152° 57.68' E | 7300.3-7055.2 | 2016-08-29 | SMF 51059 | BOLD:ADF5254 | x | MH272101 |
| AJAKK1000-17 | 40S_Rhac1_2016_6 | KBII | 40 | 45° 38.00' N | 152° 55.95' E | 45° 40.83' N | 152° 57.68' E | 7300.3-7055.2 | 2016-08-29 | SMF 51060 | BOLD:ADF5254 | x | MH272105 |

| | | | | | | | | | | | | | |
|---|---|---|---|---|---|---|---|---|---|---|---|---|---|
| AJAKK1001-17 | 40S_Rhac1_2016_7 | KBII | 40 | 45° 38.00' N | 152° 55.95' E | 45° 40.83' N | 152° 57.68' E | 7300.3-7055.2 | 2016-08-29 | SMF 51061 | BOLD:ADF5254 | x | MH272114 |
| AJAKK1002-17 | 40S_Rhac1_2016_8 | KBII | 40 | 45° 38.00' N | 152° 55.95' E | 45° 40.83' N | 152° 57.68' E | 7300.3-7055.2 | 2016-08-29 | SMF 51062 | BOLD:ADF5254 | MH272098 | MH272123 |
| AJAKK1003-17 | 40S_Rhac1_2016_9 | KBII | 40 | 45° 38.00' N | 152° 55.95' E | 45° 40.83' N | 152° 57.68' E | 7300.3-7055.2 | 2016-08-29 | SMF 51063 | BOLD:ADF5254 | x | MH272110 |
| AJAKK995-17 | 40S_Rhac1_2016_10 | KBII | 40 | 45° 38.00' N | 152° 55.95' E | 45° 40.83' N | 152° 57.68' E | 7300.3-7055.2 | 2016-08-29 | SMF 51064 | BOLD:ADF5254 | x | MH272125 |
| AJAKK946-17 | 42S_Rhac_2016_1 | KBII | 42 | 45° 39.62' N | 152° 56.39' E | 45° 40.26' N | 152° 57.63' E | 7110.6-7119.6 | 2016-08-30 | x | BOLD:ADF5254 | x | MH272108 |
| AJAKK1051-17 | 85S_Rhac1_2016_186U | KBII | 85 | 45° 02.26' N | 151° 02.14' E | 45° 01.64' N | 151° 03.68' E | 4903.4-5265.6 | 2016-09-15 | x | BOLD:ADH6163 | x | MH272124 |
| AJAKK1059-17 | 97S_Rhac1_2016_238U | KBII | 97 | 44° 05.68' N | 151° 24.88' E | 44° 06.94' N | 151° 24.88' E | 6440.4-6560.7 | 2016-09-18 | x | BOLD:ADF5254 | MH272099 | MH272115 |
| AMPIV001-17 | DZMB-HH 52103 | IceAGE | 1019/1 | 62° 56.34' N | 20° 44.64' W | x | x | 913.6 | 2011-09-03 | x | BOLD:ADH1784 | x | MG521128 |
